# The Role of the Context of Physical Activity for Its Association with Affective Well-Being: An Experience Sampling Study in Young Adults

**DOI:** 10.3390/ijerph191710468

**Published:** 2022-08-23

**Authors:** Yu-Mei Li, Justin Hachenberger, Sakari Lemola

**Affiliations:** 1 Department of Psychology, Bielefeld University, Universitätsstraße 25, 33615 Bielefeld, Germany; 2 Department of Psychology, University of Warwick, University Road, Coventry CV4 7AL, UK

**Keywords:** mental health, physical activity, exposure to nature, contextual factors, experience sampling method (ESM)

## Abstract

Physical activity and being outdoors both improve affective well-being. However, little is known about the synergistic effects between them and the influences of contextual factors such as the life domain of physical activity (work-, chores-, leisure, or sports-related) or the type of the outdoor environment (green space, blue space, or city area) on mood. This study investigates the synergistic effects of physical activity and being outdoors as well as the potential role of contextual factors on mood. A total of 158 individuals aged 18–25 years (133 females) participated in a 14-day experience sampling study. Participants received seven prompts per day and answered questions about their physical activity, contextual factors, and affective well-being. Physical activity and being outdoors were associated with concurrent higher levels of positive and lower levels of negative affect compared to being physically inactive or being indoors, respectively. However, no synergistic effects were found. Being outdoors in a city area was associated with a less positive and more negative affect than being in nature. Work- and chores-related physical activity was associated with less positive affect and more negative affect compared to sports- or leisure-related physical activity. To foster positive affect, people should schedule leisure-related physical activity in nature.

## 1. Introduction

Physical activity is beneficial to both mental and physical health—being physically active improves physical health [1,2] and physical activity as an intervention is moderately effective in treating depressive disorders [3]. An elevated mood immediately following physical activity in everyday life has been broadly reported in studies adopting experience sampling methods (ESM) [4,5,6,7,8,9,10,11,12,13,14,15,16,17,18,19,20]. The mechanism behind this association may relate to the fact that engaging in physical exercise fosters the individual’s perception of being resourceful, in control of their actions [21], and striving towards increased physical fitness and bodily resources [22,23], which in turn may contribute to affective well-being [24].

Despite the rich evidence on the mood-enhancing effects of physical activity, the role of the context in which physical activity occurs is less well-studied. The context could refer to the environment in which physical activity occurred—outdoors or indoors, in grey (e.g., cities or buildings), green (e.g., forests or parks), or blue spaces (e.g., beaches, seas, or lakes)—or the reasons why people were physically active, whether it was work-related (e.g., cycling or walking to work or doing household chores) or leisure-related (e.g., going for a walk, hiking, cycling, working out in a gym, playing sports). There is evidence that being outdoors and particularly being in green and blue spaces is related to an enhanced positive affect [25,26,27,28,29,30] and reduced negative affect [31] and stress [25,32]. The affective well-being-augmenting effect of nature may be explained by the biophilia hypothesis [33], which refers to an innate tendency to affiliate with nature. This tendency could be due to human’s evolutionary past of dwelling in nature, which provided food and shelter. However, the synergistic effects of physical activity and being outdoors has received much research interest. One recent study found independent effects of physical activity and exposure to nature but no interaction between the two [32]; stress levels measured in the evening were lower in the participants who visited a park compared to the participants who did not visit a park during the day. Regarding the reasons for physical activity, a meta-analysis of cross-sectional studies showed that self-reports of aggregated physical activity in the context of leisure, transport, and school sports were positively related to mental health and negatively associated with mental ill-health while work-related physical activity was positively related to mental ill-health [34]. This pattern of inverted affective outcomes of physical activity depending on whether it was more leisure- vs. work-related may be explained by the self-determination theory, which posits that activities imposed by external circumstances will be perceived as less enjoyable and may lead to more negative outcomes compared to self-determined activities [35]. However, and notably, the relationship between work-related physical activity and psychological distress was moderated by the type of occupation and might reflect how satisfied people are with their work [36].

Only a few studies have examined the context of physical activity by employing an ESM design. An advantage of ESM designs is that variables are measured multiple times in the same participants which allows for a study of within-individual variation of physical activity across different contexts and its relationship with within-individual variation in affect. There is evidence that inferring within-person processes based on between-individual covariation can be misleading [37]. We are aware of only one experience sampling study which examined undergraduate students across 24 h and showed that leisure-time physical activity was related to increased positive affect and calmness [12]. However, the interpretation of this study was limited by only sampling affect across one single day, which arguably may have limited within-individual variation in affect. We therefore aimed to examine the relations between the context under which physical activity occurred and the corresponding affective states. In particular, we set out to investigate whether being outdoors and being physically active were associated with people’s mood independently and whether there were synergistic effects between being outdoors and physical activity. Moreover, we examined whether leisure-related physical activity would enhance mood more than work-related physical activity. Our hypotheses were:

**H1a:**  *Being outdoors is associated with higher levels of positive affect and lower levels of negative affect compared to being indoors*;

**H1b:**  *Being physically active is associated with higher levels of positive affect and lower levels of negative affect compared to being physically inactive*;

**H1c:**  *Being outside and being physically active interact such that their synergistic effect is stronger than what would be expected based on the main effects of both variables (i.e., in terms of a higher positive affect and lower negative affect)*;

**H2:**  *The effect of being outdoors is moderated by the type of environment a person is currently in. Being in a natural environment (e.g., green or blue space) is associated with higher levels of positive affect and lower levels of negative affect than being in an artificial environment (e.g., city area)*;

**H3:**  *Work-related physical activity is associated with a lower positive affect and higher negative affect than leisure- or sports-related physical activity*.

Finally, we also studied positive and negative affect items separately in an exploratory fashion to examine whether some facets of positive or negative affect showed stronger associations with different contexts of physical activity than others. These exploratory analyses were informed by research showing that facets and nuances of emotional experience and personality may explain incremental variance in later behaviours beyond what is explained by broader traits and aggregated affect scales such as positive and negative affect or depression [38,39,40,41].

## 2. Materials and Methods

### 2.1. Procedure

This study was approved by the Ethics Committee of Bielefeld University (file no. 2020-138) and a detailed report on the procedure and methods of the study can be found in Hachenberger et al. [42], who reported on the association between daily physical activity, subsequent sleep, and affective well-being the next morning. The study population was a convenience sample. Recruitment occurred through numerous channels, including social media, word of mouth, email lists of various German universities where students can opt-in to receive information about currently running studies, and the study management portal of Department of Psychology at Bielefeld University. Participants first registered via an online questionnaire in which they were informed about the procedure and conditions of the study including data handling and protection. All participants gave informed consent. Further, participants confirmed that they were between 18 and 25 years old and that they had a smartphone with an Android operating system available for the duration of the study. Data collection took place in two batches: Batch 1 from 23 March to 6 April 2021 and Batch 2 from 30 March to 13 April 2021. Each batch lasted for 15 days and always began on a Tuesday.

On the first day of the study, participants completed a baseline questionnaire (approx. 35 min), where information regarding demographics, habitual physical activity and mental well-being were collected. On the second day, the ESM questionnaires presented on movisensXS (version 1.5.13; library version 7365; movisens GmbH, Karlsruhe, Germany) started. Participants received short questionnaires (taking approx. 2–3 min) seven times per day for 14 consecutive days (max. 98 questionnaires per participant). Participants received a prompt for the first questionnaire of the day at a random time between 8:00 a.m. and 9:00 a.m. on weekdays and between 9:00 a.m. and 10:00 a.m. on weekends. For the subsequent five questionnaires, the prompts were sent out at random times between 10:30 a.m. and 7:30 p.m. on weekdays and between 11:30 a.m. and 7:30 a.m. on weekends. For the last questionnaire of the day, prompts were sent out between 9:00 p.m. and 10:00 p.m. on all days. There was an interval of at least 90 min between prompts. After receiving a prompt, participants could respond to the questionnaires within 30 min. If participants did not respond to a questionnaire within 30 min, this questionnaire was considered as missing. Participants were instructed to ignore the prompts in situations that could cause danger to themselves or others (e.g., while driving).

Participants who completed at least 72% of all questionnaires received either a EUR 40 voucher or research participation credits. In addition, three EUR 100 vouchers were raffled among all students qualifying for compensation.

### 2.2. Participants

In total, 200 participants signed up for the study, but 42 participants did not start the study (e.g., due to personal reasons or technical incompatibility). The remaining 158 participants started the study and were included in the analyses (133 females, 23 males, and two participants who did not indicate their gender). The mean age of the sample was 22.6 years (*SD* = 2.0).

### 2.3. Measures and Instruments

#### 2.3.1. Baseline Measures

The baseline questionnaire included demographics questions as well as standardized questionnaires measuring habitual physical activity and depressive symptoms. Habitual physical activity was measured using the International Physical Activity Questionnaire (IPAQ) [43] and depressive symptoms were measured using the Patient Health Questionnaire (PHQ-9) [44,45].

#### 2.3.2. Domain of Physical Activity

In each of the seven daily questionnaires, participants were asked how physically active they have been the last 90 min and in which domain the physical activity mainly was (“*Was the physical activity you did in the last 90 min mainly in the context of...?*”). Participants could select one of the following response options: “*I haven’t been physically active*”, “*work*”, “*chores*”, “*sports*”, “*leisure time (except sports)*”, or “*other*”.

#### 2.3.3. Being Outdoors, Current Environment, and Current Activity

In the second to the sixth questionnaires of each day, participants were asked about their current location (“*Where are you at the moment?*”) and could select multiple of the following response options: “*at home*”, “*at the workplace (working from home not included)*”, “*at a university event/class/library*”, “*at the home of family/friends/partner*”, “*in a shop*”, “*outdoors*”, “*in a vehicle*”, or “*other place*”.

If the participants indicated being outdoors, they were additionally asked the type of environment they were in (“*How would you describe the place you are at?*”) with the following options from which they had to choose one: “*green spaces (e.g., in a park, forest)*”, “*near water (e.g., at a lake, sea)*”, or “*city area*”.

Furthermore, participants were asked about their current activity before receiving the prompt (“*What were you doing when you received the prompt?*”). Participants could indicate what they were working on, “work related to studies (e.g., studying)” or “*work not related to studies*”.

#### 2.3.4. Affective States

Based on Das-Friebel et al. [46], positive affect was measured with five positive items including attentive, content, enthusiastic, happy, and relaxed. Negative affect was measured with five negative items including annoyed, bored, sad, upset, and worried. These items were originally taken from the Positive and Negative Affect Scale (PANAS) [47] and Russell’s Circumplex Model of Affect [48]. At each prompt of the ESM questionnaire, participants were asked to indicate on a visual analogue scale to what extent they felt about each affective state (“*How … do you feel at the moment*?”, 0 = *not at all*, 100 = *very much*). Internal consistency was found to be good for positive affect (α = 0.84) and acceptable for negative affect (α = 0.77). Sum scores for positive and negative affect were computed separately. Higher sum scores indicate higher levels of positive and negative affect.

### 2.4. Statistical Analyses

Data pre-processing and analyses were conducted using R (version 4.2.0) [49]. A dummy variable was created for “being currently outdoors” (*no/yes*; reference category = *no*), with “*yes*” meaning that participants have selected “*being outdoors*”. Another dummy variable was created for “being physically active 90 min before receiving the prompt” (*no/yes*; reference category = *no*), with “*no*” meaning that participants did select “*I haven’t been physically active*” and “*yes*” meaning that participants selected any domain of physical activity. A third dummy variable was created for “being currently working” (*no/yes*; reference category = *no*), with “*yes*” meaning that participants indicated that they were currently working no matter whether the work was study-related. The current environment (“*indoors*”, “*city area*”, “*green space*”, or “*blue space*”) and domains of physical activity (“*no physical activity*”, “*work*”, “*chores*”, “*sports*”, “*leisure*”, or “*other*”) were included as categorical variables in the respective models.

Multilevel models were used to test the hypothesised associations. Computation of the multilevel models was performed with the R-package lme4 (version 1.1-29) [50] using maximum likelihood estimation. Positive and negative affect were treated as separate outcome variables for all the hypotheses. To test H1a–c, the dummy variables “being outdoors” and “physical activity during the 90 min before the prompt” and the interaction between the two variables were used as predictors. In addition, the covariate of “working” vs. “not working” at the time when answering the questionnaire was added to the models to test how it affected the associations tested in H1a–c. To test H2 and H3, models with the current environment and the domain of physical activity were included as predictors, respectively. For pairwise comparisons, contrasts of estimated marginal means of respective categories were computed. A random intercept for each participant was included in all models.

For exploratory purposes, post-hoc analyses were conducted to investigate single affect items separately. Ten separate models were used to test each of the ten affect items as outcome variables. However, in the post-hoc analyses, green and blue areas and sports-related and leisure-related physical activity were combined as categories when examining the contrasts.

To focus on the within-individual effects and to make the effects comparable, positive and negative affect sum scores were within-individual standardised before analysis. The significance threshold was set to 0.05. All obtained *p*-values were adjusted using false discovery rate (FDR) [51] to address the problem of alpha error accumulation.

## 3. Results

### 3.1. Descriptive Statistics

On average, participants responded to 78.2% of all prompts, resulting in 12,108 assessments in total. Based on the participants’ responses to the IPAQ at baseline, 113 (71.5%) completed 150 min of moderate-intensity activity per week, or 75 min of vigorous-intensity activity per week, or an equivalent combination of moderate- and vigorous-intensity physical activity as recommended, e.g., by the Physical Activity Guidelines for Americans [52]. Concerning depressive symptoms, the mean PHQ-9 score was 7.7 (*SD* = 5.1). A total of 51 participants (32.3%) were classified as having no/minimal depressive symptoms, 63 (39.9%) with mild depressive symptoms, and 44 (27.9%) with moderate-severe depressive symptoms. The descriptive statistics for the dependent variables are displayed in Table 1.

Across all available assessments, participants were mostly indoors (93.2%; outdoors 6.8%). While being outdoors, participants were mainly in green spaces (58.2%; i.e., forest or park), followed by city areas (34.7%), and blue spaces (7.1%; i.e., lake or river). Before most assessments, participants were physically inactive (66.4%), followed by being physically active in the domain of leisure (excluding sports) (11.3%), chores (10.0%), sports (4.6%), other (5.0%), and work (2.7%). 

### 3.2. Association of Physical Activity and Being Outdoors with Affective States

**Figure 1:**
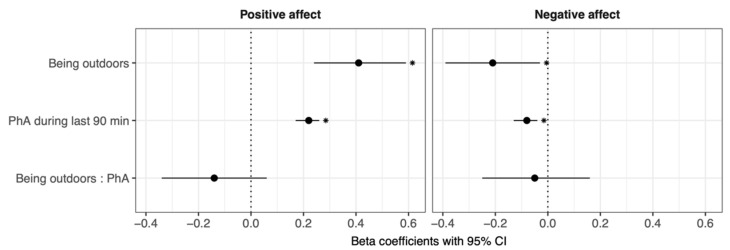
Associations of being outdoors and being physically active with affective states. PhA = physical activity. Asterisks indicate that the respective association was significant after FDR-adjustment.

Multilevel models testing the associations of H1a–c (Figure 1) revealed that being outdoors was associated with higher positive affect (β = 0.41, *p* < 0.001) and lower negative affect (β = −0.21, *p* < 0.05) compared to being indoors (H1a). Moreover, being physically active during the last 90 min was associated with higher positive affect (β = 0.22, *p* < 0.001) and lower negative affect (β = −0.08, *p* < 0.001) compared to not being physically active (H1b). No significant interaction of being outside and physical activity during the last 90 min was found for positive or negative affect (H1c).

To test the effects of being at work or having leisure time while completing the questionnaire, we ran further models including working vs. leisure time as a covariate. When “working at the time of completing the questionnaire “ was included as a covariate, we found that being outdoors was still associated with higher positive affect (β = 0.38, *p* < 0.001) compared to being indoors (H1a). However, no significant association with negative affect (β = −0.18, *p* = 0.075, *p.unadjusted* = 0.049) was found after FDR correction. Being physically active during the last 90 min was still associated with higher positive affect (β = 0.19, *p* < 0.001) and lower negative affect (β = −0.06, *p* < 0.05) compared to not being physically active (H1b).

#### 3.2.1. Pairwise Comparisons of Different Outdoor Environments with Affective States

**Figure 2:**
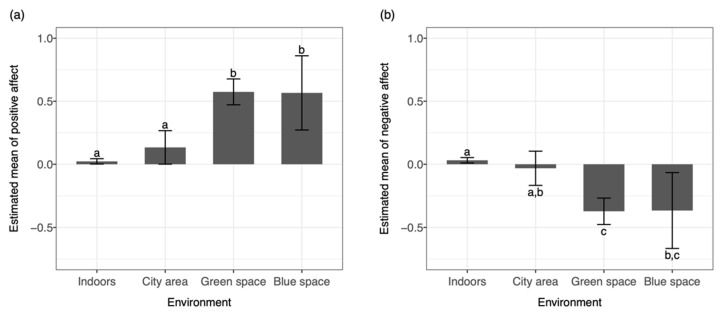
Pairwise comparisons of the estimated marginal means of (**a**) positive affect and (**b**) negative affect between different environments. Domains with different superscripts are statistically different from each other (*p* < 0.05).

The pairwise comparisons of different types of outdoor environments and their association with affective states (H2) revealed that being outdoors in a city area was associated with lower positive affect (β = −0.44, *p* < 0.001) and higher negative affect (β = 0.34, *p* < 0.001) compared to being in a green outdoor space. Moreover, being outdoors in a city area was associated with a lower positive affect (β = −0.43, *p* < 0.05) but not with a higher negative affect compared to being in a blue outdoor space. No significant differences between being in a green and blue outdoor space were observed. All pairwise comparisons are presented in Table 2 and Figure 2.

#### 3.2.2. Pairwise Comparisons of Different Domains of Physical Activity with Affective States

The pairwise comparison testing the differences in affective states between work- and leisure-related physical activity (H3) revealed that work-related physical activity was associated with a lower positive affect (β = −0.37, *p* < 0.001) and a higher negative affect (β = 0.25, *p* < 0.001) compared to the leisure-related physical activity. Moreover, work-related physical activity was associated with a lower positive affect (β = −0.43, *p* < 0.001) and a higher negative affect (β = 0.31, *p* < 0.001) compared to sports-related physical activity.

Similarly, chore-related physical activity was associated with a lower positive affect (β = −0.11, *p* < 0.01) and a higher negative affect (β = 0.17, *p* < 0.001) compared to a leisure-related physical activity. Moreover, chore-related physical activity was associated with a lower positive affect (β = −0.18, *p* < 0.01) and a higher negative affect (β = 0.24, *p* < 0.001) compared to sports-related physical activity.

**Figure 3:**
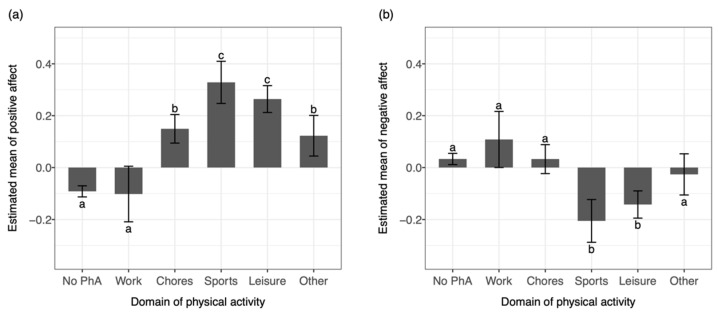
Pairwise comparisons of the estimated marginal means of (**a**) positive affect and (**b**) negative affect between different domains of physical activity. Domains with different superscripts are statistically different from each other (*p* < 0.05).

There were no significant differences between leisure- and sports-related physical activity for positive and negative affect. However, work-related physical activity was also associated with a lower positive affect (β = −0.25, *p* < 0.001) but not with a negative affect compared to chores-related physical activity. All pairwise comparisons are presented in Table 3 and Figure 3.

### 3.3. Post-Hoc Analyses of the Associations of Single Affect Items with Being Outdoors and Physical Activity

Concerning the single positive affect items, it was found that being outdoors was associated with being more attentive (β = 0.21, *p* < 0.05), content (β = 0.31, *p* < 0.01), enthusiastic (β = 0.27, *p* < 0.01), happy (β = 0.40, *p* < 0.001), and relaxed (β = 0.31, *p* < 0.01). However, being outdoors was not significantly associated with any of the single negative affect items.

**Figure 4:**
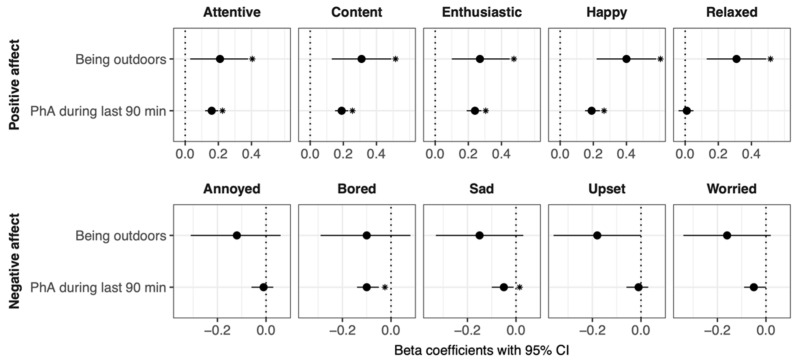
Associations of being outdoors and being physically active in the last 90 min with single affect items. PhA = physical activity. Asterisks indicate that the respective association was significant after FDR-adjustment.

Concerning the single positive affect items, it was found that being physically active during the last 90 min was associated with being more attentive (β = 0.16, *p* < 0.001), content (β = 0.19, *p* < 0.001), enthusiastic (β = 0.24, *p* < 0.001), and happy (β = 0.19, *p* < 0.001). No significant association was found for being relaxed. Concerning the single negative affect items, it was found that being physically active during the last 90 min was associated with being less bored (β = −0.10, *p* < 0.001) and sad (β = −0.05, *p* < 0.05). No significant association was found for being annoyed, upset, or worried. All associations of single affect items with being outdoors and physical activity are displayed in Figure 4.

#### 3.3.1. Pairwise Comparisons of Different Outdoor Environments with Single Affect Items

Concerning the single positive affect items, being outdoors in a city area was associated with feeling less content (β = −0.39, *p* < 0.001), enthusiastic (β = −0.34, *p* < 0.001), happy (β = −0.46, *p* < 0.001), and relaxed (β = −0.38, *p* < 0.001) compared to being in a natural (green/blue) space, which was analysed as a combined category in these post-hoc analyses. No differences were found for feeling attentive.

Concerning the single negative affect items, being outdoors in a city area was associated with feeling more annoyed (β = 0.32, *p* < 0.001) and worried (β = 0.32, *p* < 0.001) compared to being in a natural space. No differences were found for the other negative affect items.

#### 3.3.2. Pairwise Comparisons of Different Domains of Physical Activity with Single Affect Items

Concerning the single positive affect items, work-related physical activity was associated with feeling less content (β = −0.36, *p* < 0.001), enthusiastic (β = −0.26, *p* < 0.001), happy (β = −0.35, *p* < 0.001), and relaxed (β = −0.46, *p* < 0.001) than sports-/leisure-related physical activity, which was also analysed as a combined category in these post-hoc analyses. No differences were found for feeling attentive.

Concerning the single negative affect items, work-related physical activity was associated with feeling more annoyed (β = 0.32, *p* < 0.001) and bored (β = 0.30, *p* < 0.001) than sports-/leisure-related physical activity. No differences were found for the other negative affect items.

## 4. Discussion

Our findings show that being outdoors and being physically active were associated with an increased positive affect and decreased negative affect. Generally, the effect-sizes were considerably larger for the effects related to positive affect than for negative affect. In direct comparisons, spending time in the city area elicited a less positive affect and more negative affect than spending time in nature. Moreover, work- and chore-related physical activity was linked to lower positive affect and more negative affect compared to leisure- and sports-related physical activity. Furthermore, work-related physical activity was associated with decreased positive affect compared to chore-related physical activity. Importantly, while we found main effects of physical activity and being outdoors, there was no significant interaction between the two. Thus, the effects of physical activity and being outdoors appeared to be additive and no synergistic increase or decrease was present.

In exploratory item-level analyses, we found that people were more content, enthusiastic, and happy when they spent time in nature and/or were physically active for sports- or leisure purposes than when they were physically inactive, doing work-related physical activity, or spending time indoors or outdoors in the city areas. People felt more relaxed when spending time in nature and less relaxed when they were in the city area or while being physically active for work-related tasks. After physical activity, people were less bored and sad, while work-related physical activity was associated with boredom and annoyance. Similarly, being in the city area was associated with feeling more worried and annoyed than in nature.

Our results are in line with the previous research in that positive mood was increased and negative mood was decreased after physical activity [4,5,6,7,8,9,10,11,12,13,14,15,16,17,18,19,20] and while being outdoors [25,26,27,28,29,30,31]. Using ESM data and applying between-individual analysis, Park et al. found no interaction between physical activity and being outdoors [32], which was also consistent with our results studying within-individual variation. Leisure- and sports-related physical activity were associated with more favourable affective states than work-related physical activity, which is also also consistent with previous research [34],.

Our findings are also in agreement with the biophilia hypothesis, which proposes that human beings prefer being around plants and feel better in natural surroundings compared to being surrounded by indoor or urban environments [33]. As the reason for this effect, it has been suggested that in the times of our hunter–gatherer ancestors, plants provided food, shelter, and possibilities to hide, while water provided the opportunity to drink, wash oneself, and cool oneself down on hot days [53]. Thus, the positive effects of natural environments on mood have been supposed to be due to an inborn preference of environments that signal increased evolutionary adaptedness. In a similar vein, engaging in physical activity for sports and leisure purposes, and thereby affording to spend spare energy, may signal to one-self and others that one is resourceful [21]. Furthermore, the expectation of the physical training effect and thereby the build-up of new bodily resources in terms of increased physical fitness may lead to a self-perception of personal flourishing, which is defined as the experience of actively building up resources and that life is going well [22,23]. Work- and chore-related physical activity were associated with a less positive affect compared to sports- and leisure-related physical activity. However, the difference to sports- and leisure-related physical activity concerning positive affect was smaller for chore-related physical activity than for work-related physical activity. This finding is in line with predictions of the self-determination theory [35] in that highly self-determined physical activity (i.e., physical activity for sports and leisure purposes) was associated with increased positive and decreased negative affect while physical activity with a lower degree of self-determination (i.e., physical activity for work purposes and doing chores), was less likely to elicit a positive affect and was even related to an increased likelihood to experience boredom and annoyance.

The associations between single affective states with physical activity and being outdoors shows that the effects of physical activity and exposure to nature were associated with the same affective states. This means that their contribution to affective well-being does not tap into different aspects of mood but rather they have uniform effects on the experience of being happy and content. Only with regard to “feeling relaxed” differential associations in terms of significance and effect size were observed. While being outdoors was related to being more relaxed, no such effect was found for physical activity. The lack of an effect of physical activity on “feeling relaxed” might reflect the effects of competitive sports which are both mentally and physically activating for most people at least as an acute effect. However, this was not tested here and replications of the item-level associations are needed to reach more reliable and solid conclusions.

Experiencing positive affect in every-day life is highly beneficial as it increases resilience against illness, e.g., related to infections [54] and poor mental health in general [55]. As described by the broaden-and-build theory [55], positive emotions facilitate a state of mind of broadened perspective involving creative and unconstrained thinking and acting, which motivates the build-up of new psycho-social resources including new social relationships or skills as well as economic resources [55]. Our findings may inform the design of interventions that aim to facilitate people spending more time on such a broaden-and-build-state of mind. On a personal level, physical activity and time spent in nature could for instance be scheduled on multiple occasions across the day [4] and research may determine the benefit of different schemes, which involve varying levels of activity in different settings and varying degrees of perceived self-determination. On a societal level, the design of urban areas, workplaces, and work schedules may enable people to satisfy their need for self-determined physical activity and time in nature [56].

Our study is not without limitations. First, we adopted an observational design, which precludes drawing causal conclusions. Second, the domain-specific physical activity measured in our study only covered one type–either work-, chore-, leisure-, or sports-related physical activity during the past 90-min period of each assessment. Therefore, it could not capture the instances when people performed more than one type of the four domains. Future studies could simultaneously measure various domains of physical activity happening at the same time periods to gain a comprehensive view of physical activity and their associations with mood. Third, our design does not consider that the physical activity happened before the preceding 90 min and the potential influences of this on mood. Fourth, we did not measure physical activity objectively by accelerometry. Fifth, our sample was not representative of the general population and by including predominantly young university students and there were more women than men. Finally, the data were collected during a COVID-19-related partial lockdown in Germany, which had been ongoing for months. This could have amplified the association of positive affect with being outdoors vs. being indoors. Moreover, being outdoors could have been a planned activity rather than a circumstance due to people’s daily routines (e.g., going to university, walking to another building).

## 5. Conclusions

In conclusion, physical activity and being outdoors were linked to increases in positive affect and, to a somewhat lesser degree, decreases in negative affect. In particular, these associations were more noticeable in nature vs. in city area and leisure- and sports-related physical activity vs. work- and chore-related physical activity. Our results may inform the design of interventions involving scheduled leisure-related physical activity in nature.

## Figures and Tables

**Table 1:** Descriptive statistics for the affective state sum scores and single affect items.

	*M*	*SD*	Observed Range
Positive affect	287.9	90.1	0.0–500.0
Attentive	55.6	22.7	0.0–100.0
Content	63.8	22.2	0.0–100.0
Enthusiastic	47.0	24.9	0.0–100.0
Happy	63.7	22.1	0.0–100.0
Relaxed	58.0	23.5	0.0–100.0
Negative affect	102.5	83.9	0.0–478.0
Annoyed	23.2	25.7	0.0–100.0
Bored	21.0	22.0	0.0–100.0
Sad	17.9	22.0	0.0–100.0
Upset	14.8	20.4	0.0–100.0
Worried	25.6	25.4	0.0–100.0

**Table 2:** Pairwise comparisons of affective states between different environments.

Outcome	Contrast	β	*SE*	*t*	*p*
Positive affect	Indoors–city area	−0.11	0.07	−1.61	0.149
	Indoors–green space	−0.55	0.05	−10.32	<0.001
	Indoors–blue space	−0.54	0.15	−3.61	<0.01
	City area–green space	−0.44	0.09	−5.14	<0.001
	City area–blue space	−0.43	0.16	−2.62	<0.05
	Green–blue space	0.01	0.16	0.05	0.985
Negative affect	Indoors–city area	0.06	0.07	0.90	0.422
	Indoors–green space	0.40	0.05	7.41	<0.001
	Indoors–blue space	0.40	0.15	2.59	<0.05
	City area–green space	0.34	0.09	3.90	<0.001
	City area–blue space	0.33	0.17	1.99	0.072
	Green–blue space	−0.01	0.16	−0.04	0.985

**Table 3:** Pairwise comparisons of affective states between different domains of physical activity.

Outcome	Contrast	β	*SE*	*t*	*p*
Positive affect	No PhA–Work	0.01	0.06	0.18	0.893
	No PhA–Chores	−0.24	0.03	−7.99	<0.001
	No PhA–Sports	−0.42	0.04	−9.79	<0.001
	No PhA–Leisure	−0.36	0.03	−12.44	<0.001
	No PhA–Other	−0.21	0.04	−5.17	<0.001
	Work–Chores	−0.25	0.06	−4.09	<0.001
	Work–Sports	−0.43	0.07	−6.28	<0.001
	Work–Leisure	−0.37	0.06	−6.04	<0.001
	Work–Other	−0.22	0.07	−3.32	<0.01
	Chores–Sports	−0.18	0.05	−3.58	<0.01
	Chores–Leisure	−0.11	0.04	−2.97	<0.01
	Chores–Other	0.03	0.05	0.55	0.652
	Sports–Leisure	0.06	0.05	1.31	0.240
	Sports–Other	0.21	0.06	3.57	<0.01
	Leisure–Other	0.14	0.05	2.95	<0.01
Negative affect	No PhA–Work	−0.08	0.06	−1.34	0.232
	No PhA–Chores	0.00	0.03	0.01	0.991
	No PhA–Sports	0.24	0.04	5.49	<0.001
	No PhA–Leisure	0.18	0.03	6.05	<0.001
	No PhA–Other	0.06	0.04	1.41	0.215
	Work–Chores	0.08	0.06	1.22	0.270
	Work–Sports	0.31	0.07	4.52	<0.001
	Work–Leisure	0.25	0.06	4.09	<0.001
	Work–Other	0.13	0.07	1.97	0.074
	Chores–Sports	0.24	0.05	4.69	<0.001
	Chores–Leisure	0.17	0.04	4.48	<0.001
	Chores–Other	0.06	0.05	1.19	0.279
	Sports–Leisure	−0.06	0.05	−1.27	0.254
	Sports–Other	−0.18	0.06	−3.07	<0.01
	Leisure–Other	−0.12	0.05	−2.39	<0.05

PhA = physical activity.

## Data Availability

The data presented in this study are available on request from the corresponding author. The data are not publicly available due to privacy or ethical restrictions.
